# Artificial intelligence in improving the outcome of surgical treatment in colorectal cancer

**DOI:** 10.3389/fonc.2023.1116761

**Published:** 2023-01-17

**Authors:** Mihaela Flavia Avram, Daniela Cornelia Lazăr, Mihaela Ioana Mariş, Sorin Olariu

**Affiliations:** ^1^ Department of Surgery X, 1st Surgery Discipline, “Victor Babeş” University of Medicine and Pharmacy Timişoara, Timişoara, Romania; ^2^ Department of Mathematics, Politehnica University Timisoara, Timişoara, Romania; ^3^ Department V of Internal Medicine I, Discipline of Internal Medicine IV, “Victor Babeş” University of Medicine and Pharmacy Timişoara, Timişoara, Romania; ^4^ Department of Functional Sciences, Division of Physiopathology, “Victor Babes” University of Medicine and Pharmacy Timisoara, Timisoara, Romania; ^5^ Center for Translational Research and Systems Medicine, “Victor Babes” University of Medicine and Pharmacy Timisoara, Timisoara, Romania

**Keywords:** artificial intelligence, colorectal cancer, automated robotic surgery, phase recognition, excision plane navigation, endoscopy control, annotated video banks

## Abstract

**Background:**

A considerable number of recent research have used artificial intelligence (AI) in the area of colorectal cancer (CRC). Surgical treatment of CRC still remains the most important curative component. Artificial intelligence in CRC surgery is not nearly as advanced as it is in screening (colonoscopy), diagnosis and prognosis, especially due to the increased complexity and variability of structures and elements in all fields of view, as well as a general shortage of annotated video banks for utilization.

**Methods:**

A literature search was made and relevant studies were included in the minireview.

**Results:**

The intraoperative steps which, at this moment, can benefit from AI in CRC are: phase and action recognition, excision plane navigation, endoscopy control, real-time circulation analysis, knot tying, automatic optical biopsy and hyperspectral imaging. This minireview also analyses the current advances in robotic treatment of CRC as well as the present possibility of automated CRC robotic surgery.

**Conclusions:**

The use of AI in CRC surgery is still at its beginnings. The development of AI models capable of reproducing a colorectal expert surgeon’s skill, the creation of large and complex datasets and the standardization of surgical colorectal procedures will contribute to the widespread use of AI in CRC surgical treatment.

## 1 Introduction

Colorectal cancer (CRC) is the second most prevalent cause of cancer-related deaths worldwide and the third most common malignancy in both men and women, respectively (1, 2),. With liver metastases present in nearly 20% of cases, 60–70% of individuals with clinical symptoms of CRC are detected at advanced stages. Additionally, individuals with metastatic dissemination at the time of diagnosis had a 5-year overall survival rate of only 10-15%, compared to patients with local malignancy, which ranges from 80-90% (3).

Artificial intelligence (AI) is a branch of computer science that focuses on creating intelligent computers capable of performing activities that normally necessitate human intelligence. Several Ai technologies exist all around us, but understanding and evaluating their impact on today’s society might be difficult. Deep learning algorithms and support vector machines (SVMs) have made important contributions to this advanced technology during the last decade, playing a key role in medical and healthcare systems (4).

There are two types of AI applications in the medical field: virtual and physical. The virtual component of AI is made up of machine learning (ML) and deep learning (DL, a subset of ML) (5). There are three types of machine learning algorithms: supervised, unsupervised, and reinforcement learning. Meanwhile, the most well-known deep learning scheme, a convolutional neural network (CNN), is a sort of multilayer artificial neural network that is extremely efficient for image categorization (6).

Artificial neural networks (ANNs) are ML tools. In function, they mimic the human brain by connecting and discovering complicated relationships and patterns in data. ANNs are made up of numerous computational units (neurons) that accept inputs, execute calculations, and send output to the next computational unit. The input is processed as signals by layers of algorithms, which produce specific patterns as final output, which are interpreted and employed in decision-making. Simple 1- or 2-layered neural networks are typically used in ANNs (7, 8).

Computer vision (CV) is focused on how computers may learn to understand digital images and videos (such as object and scene recognition) at a high level, in a manner similar to the human eye. 2 The processed data can include video sequences, several camera perspectives, or multidimensional data from a medical scanning instrument (7, 9–11).

The physical branch of AI includes medical devices and robots, such as the Da Vinci Surgical System (Intuitive Surgical Inc., Sunnyvale, CA, USA), as well as nanorobots.

A considerable number of recent research have used AI in the area of CRC (7–9). From the standpoint of clinical practice, the available AI applications in CRC primarily contain four clinical aspects (10):

- Screening: Endoscopy is the gold standard for CRC screening. AI-assisted colonoscopy for polyp detection and characterization, risk prediction models using clinical and omics data, are expected to improve CRC screening.
- Diagnosis: The qualitative diagnosis and staging of CRC are mostly based on imaging and pathological examination. DL can greatly increase medical image interpretation, minimize disparities in experience, and reduce misinterpretation rates thanks to powerful image recognition processing technology (9).
- Treatment: The treatment of CRC mainly consists of surgery, chemotherapy and radiotherapy. Novel therapies can be evaluated with the help of AI, while AI can provide a more precise treatment choice, individually tailored on each patient (11).
- Prognosis: Predicting the recurrence and estimating survival is more accurate using ML approach, as it uses various multidimensional information. Deep learning has been demonstrated to be as good as or better than statistical methods (eg. COX regression model) in cancer prognosis (12).

Surgical treatment of CRC still remains the most important curative component. Artificial intelligence in CRC surgery is not nearly as advanced as it is in screening (colonoscopy), diagnosis and prognosis. This is most likely due to the increased complexity and variability of structures and elements in all fields of view, as well as a general shortage of equivalent annotated video banks for utilization (13, 14).

The aim of this minireview is to summarize up to date information on the possibility of using AI in improving the outcome of surgical treatment of CRC. It concentrates on the intraoperative steps which can benefit from AI and summaries the published studies, it gives a brief outline of current AI applications in colorectal surgery. It also analysis the current advances in CRC robotic treatment, especially automated surgeries. In order to make appropriate decisions on topics deserving of further investigation, it is necessary to understand the existing situation of AI in the surgical treatment of CRC.

## 2 Methods

**Figure 1:**
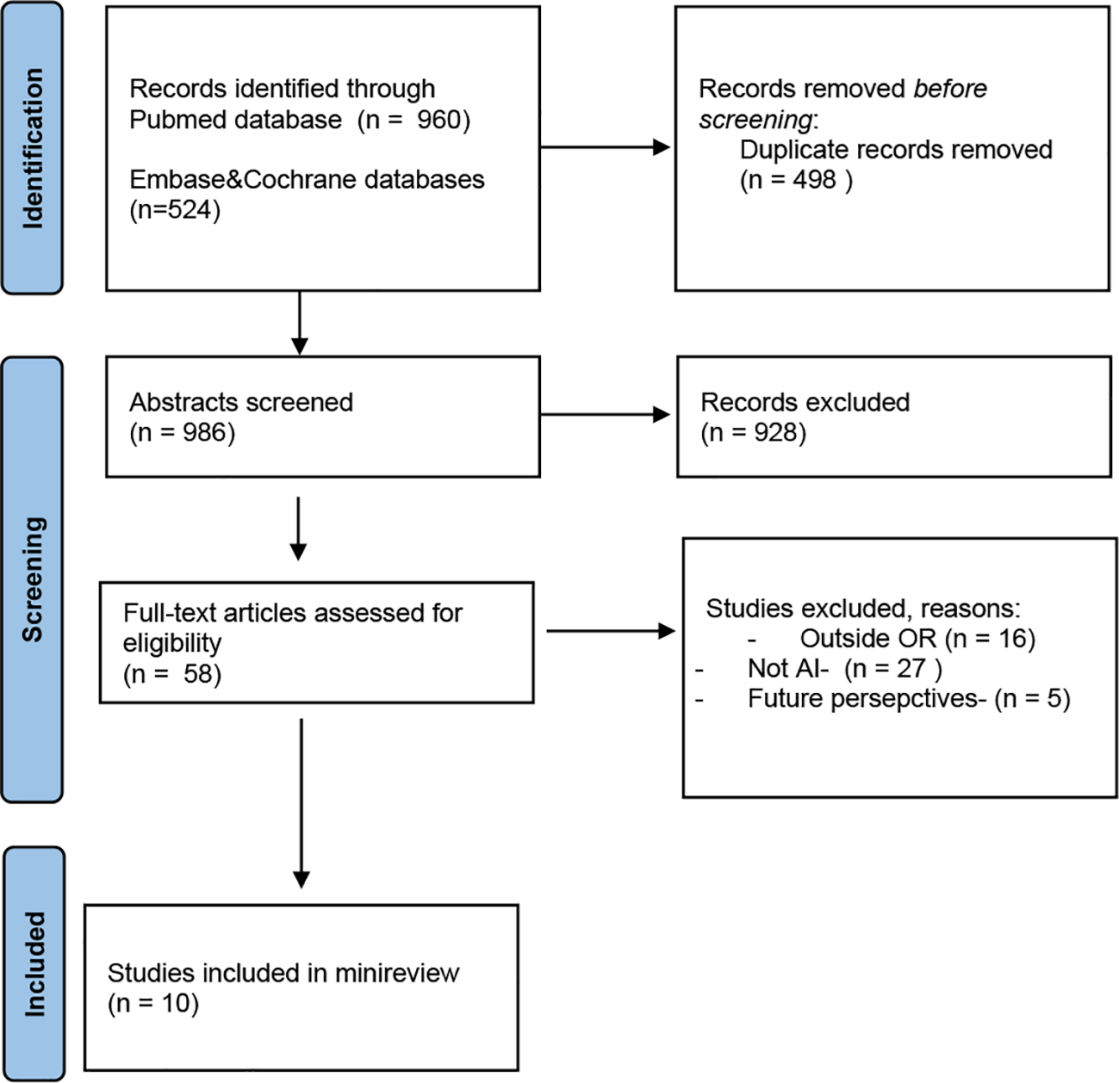
PRISMA 2020 flow diagram search (15).

A literature search was performed up to September 5th, 2022 using the following online databases: PubMed,Embase, Cochrane Library. The terms AI, OR, and surgery, including synonyms or equivalent terms, were used to obtain the literature. We have read the abstracts and selected the articles presenting data which can be used during CRC surgical treatment. The literature search retrieved 1484 articles, from 3 databases. Finally, 10 studies were included. The flow diagram can be viewed in **Figure 1**.

**Table 1** shows an overview of the included studies, their application, and the specific AI subfield the application is based on.

**Table 1:** Overview of included studies with specific applications.

Application	Study	Year	AI subfield
Autonomous robotic intestinal anastomosis	Shademan et al. (16) Saeidi et al. (17)	2016 2022	CNN
Phase recognition	Kitaguchi et al. (18)	2020	CNN
Excision plane navigation	Igaki et al. (19)	2022	DL
Camera guidance	Wagner et al. (20)	2021	ML
AI real-time microcirculation analysis using ICG	Park et al. (21)	2020	ML
Knot-tying	Weede et al. (22)	2011	RNN
Optical biopsy	Jansen-Winlem et al. (23) Collins et al. (24) Okamoto et al. (25)	2021 2022 2022	CNN CNN CNN &DL

ICG, indocyanine green; HIS, hyperspectral imaging; CNN, convolutional neural networks; DL, deep learning; ML, machine learning; RNN, recurrent neural networks.

## 3 Results

### 3.1 Surgical robots

With the introduction of robotic colorectal surgery, colorectal cancer surgical treatment has entered a new era. The da Vinci System is now the most extensively utilized robotic surgical. It allows surgeons to execute extremely delicate or highly complex procedures with wristed devices that have seven degrees of freedom. When compared to traditional open surgery, the advantages of surgery with these robots include a shorter period of recovery and hospital stay, minimum scarring, smaller incisions, and a significant reduction in the risk of surgical site infections, postoperative pain, and blood loss (26, 27). Surgeons can operate with a larger viewing field thanks to computer-controlled instruments. Computer-controlled devices enable surgeons to work with a wider viewing field, greater flexibility, dexterity, precision, and less fatigue. The da Vinci dual-console provides integrated teaching and supervision, for residents’ surgical training. The Senhance surgical robot (TransEnterix Surgical Inc., Morrisville, NC, USA) is a laparoscopic-based technology that allows skilled laparoscopic surgeons to perform more sophisticated surgeries (28).

The robotic platform has a distinct benefit in that it allows access to difficult-to-reach locations, such as a narrow pelvis, while also preserving postoperative urinary and sexual function (29). Rectal surgery was related with higher conversion rates in men, obese patients, and patients getting a low anterior resection compared to robotic surgery in the ROLARR randomized clinical study (30). Recent research has also showed that robotic-assisted surgery appears to be more suitable for protecting the pelvic autonomic nerve (29, 31, 32).

The Intuitive Surgical da Vinci system pioneered the notion of transparent teleoperation, in which motions done by the surgeon on the control interface are precisely copied by surgical tools on the patient side. The lack of a decision-making process by the machine in the transparent teleoperation paradigm gives the surgeon unlimited control. However, these devices have certain algorithmic autonomy, such as tremor suppression and redundancy resolution, which do not interfere with the surgeon’s actions (33). They can not be considered AI driven devices, but they offer a starting point for the hardware for future autonomous operating robots.

### 3.2 Autonomous surgical robots in CRC surgery

Shademan et al. described complete *in vivo* autonomous robotic anastomosis of porcine intestine utilizing the Smart Tissue Autonomous Robot (STAR) (16). STAR surpassed human surgeons in a range of ex vivo and *in vivo* surgical tasks, despite being conducted in a carefully controlled experimental context. In later *in vivo* tests, STAR obtained 66.28% correctly placed stitches in the first attempt, which corresponded to an average of 0.34 suture hesitancy per stitch (17).

For the first time, these experiments proved the fledgling clinical feasibility of an autonomous soft-tissue surgical robot. STAR was controlled by artificial intelligence (AI) algorithms and received input from an array of optical and tactile sensors, as opposed to traditional surgical robots, which are managed in real time by people and have become ubiquitous in specific subspecialties.

### 3.3 Phase and action recognition

Phase recognition is the task of identifying surgical images according to preset surgical phases. Phases are parts of surgical operations that are required to finish procedures successfully. They are often determined by consensus and recorded on surgical videos (34).

There are several studies of phase and action recognition, on different types of surgery, including colorectal surgery. The study of Kitaguchi et al. aimed to create a large annotated dataset containing laparoscopic colorectal surgery videos and to evaluate the accuracy of automatic recognition for surgical phase, action, and tool by combining AI with the dataset. They used 300 intraoperative videos and 82 million frames were marked for a phase and action classification task, while 4000 frames were marked for a tool segmentation task. 80% of the frames, were used for the training dataset and 20% for the test dataset. CNN was utilized to analyze the videos. The accuracy for the automatic surgical phase task was 81%, while the accuracy for action classification task was 83.2% (18).

### 3.4 Excision plane navigation

The creation of an image-guided navigation system for areolar tissue in the complete mesorectal excision plane using deep learning has been reported by Igaki et al. This could be useful to surgeons since areolar tissue can be utilized as a landmark for the optimum dissection plane. Deep learning-based semantic segmentation of areolar tissue was conducted in the whole mesorectal excision plane. The deep learning model was trained using intraoperative images of the whole mesorectal excision scene taken from left laparoscopic resection movies. Six hundred annotation images were generated from 32 videos, with 528 photos used in training and 72 images used in testing. The established semantic segmentation model helps in locating and emphasizing the areolar tissue area in the whole mesorectal excision plane (19).

### 3.5 Endoscopy Control

There are commercial systems available that allow the endoscopic camera to move without human intervention, following particular features in the scene., Viki (35), FreeHand (36), SOLOASSIST (37)and AutoLap (20), for example, do camera stabilization and target tracking. These were the first autonomous systems used to assist with MIS intervention. The autonomy is implemented *via* feature tracking algorithms that maintain the surgical instrument in the endoscope’s visual field (38).

For AutoLap minimally invasive rectal resection with entire mesorectal excision was chosen to experimentally test cognitive camera guidance as this surgical method places great demands on camera control. A single surgeon performed twenty surgeries with human camera guidance for learning purposes. After the completion of the surgeon’s learning curve, two different robots were trained on data from the manual camera guiding, followed by using one robot to train the other. The performance of the cognitive camera robot improved with experience The duration of each surgery improved as the robot became more experienced, also the quality of the camera guidance (evaluated by the surgeon as good/neutral/poor) improved, becoming good in 56.2% of evaluations (20).

### 3.6 AI-based real-time microcirculation analysis

In order to predict anastomotic complications attributable to hypoperfusion after laparoscopic colonic surgery, a fluorescence laparoscopic system can be used during surgery for angiography using indocyanine green (ICG). Each patient has a different perfusion status, due to individual variations in collateral circulation blood flow pathways, which provides a different ICG curve. A well-trained AI can forecast the probability of hypoperfusion-related anastomotic problems by analyzing the microcirculation state, by using numerous metrics and ICG curve patterns. The AI-based micro perfusion analysis system can help surgeons by quickly performing real-time analysis and giving information in a color map to surgeons. Using a neural network that imitate the visual cortex, Park et al. clustered 10,000 ICG curves into 25 patterns using unsupervised learning, an AI training approach that does not require annotations during training. ICG curves were derived from 65 processes. Curves were preprocessed to minimize the degradation of the AI model caused by external factors such light source reflection, background, and camera movement. The AI model revealed more accuracy in the microcirculation evaluation when the AUC of the AI-based technique was compared to T1/2 max max (time from first fluorescence increase to half of maximum), TR (time ratio: T1/2 max/Tmax, Tmax is the time form first fluorescence increase to maximum), and RS (rise slope), with values of 0.842, 0.750, 0.734, and 0.677, respectively. This makes it easier to create a color mapping scheme of red-green-blue areas that classifies the degree of vascularization. In comparison to a surgeon’s solely visual inspection, this AI model delivers a more objective and accurate approach of fluorescence signal evaluation. It can provide an immediate evaluation of the grade of perfusion during minimally invasive colorectal procedures, allowing for early detection of insufficient vascularization (21, 39).

### 3.7 Knot-tying

Knot-tying is part of basic surgical skills and a quick technique in open surgery, while laparoscopic knot-tying can take up to three minutes for a single knot to be done. Mayer et al. described a solution based on RNNs to speed up knot-tying in robotic cardiac surgery. The surgeon inputs a sequence to the network (for example, instances of human-performed knot-tying), and an RNN with long-term storage learns the task. The preprogrammed controller was able to construct a knot in 33.7 seconds, however the introduction of an RNN offered a speed improvement of about 25% after learning from 50 prior runs, generating a knot in 25.8 seconds (22, 40, 41).

### 3.8 AI in automatic optical biopsy and hyperspectral imaging for CRC

Optical biopsy is a light-based nondestructive *in situ* assessment of tissue pathologic features. Hyperspectral imaging (HSI) is a non-invasive optical imaging tool that provides pixel-by-pixel spectroscopic and spatial information about the investigated area. Tissue-light interaction produces distinct spectral signatures, allowing the visualization of tissular perfusion and differentiation of tissue types. HSI cameras are commercially available and are easily compatible with laparoscopes (42, 43).. In the past years several very promising studies, which used different AI methods in detecting CRC during surgery using HIS, were published.

Jansen-Winkeln et al. used HSI records from 54 patients who underwent colorectal resections, creating a realistic intraoperative setting for their study. By using a CNN method, they obtained a sensitivity if 86% and specificity of 95% for the distinction between cancer and healthy mucosa, while differentiating cancer against adenoma had a sensitivity of 68%, and 59% specificity o for CCR (23).

Collins et al. used HIS imaging on specimens obtained immediately after extraction from 34 patients undergoing surgical resection for CRC. Using a CNN to automatically detect CRC in the HIS images they obtained a sensitivity of 87% and specificity of 90% for cancer detection. Their approach could be used for objectively assessing tumor margins during surgery (24).

By combining HSI and CNN trained with deep learning on porcine models, Okamoto et al. obtained an automatic distinction of different anatomical layers in CRC surgery, achieving a recognition sensitivity of 79.0 ± 21.0% for the retroperitoneum and 86.0 ± 16.0% for the colon and mesentery (25)..These results are promising in improving the results of complete mesocolic excision, by lowering the complications associated with it (like lesions of the ureter, gonadal vessels)and offering a better oncologic result.

## 4 Discussions

This minireview offers an overview of various AI applications currently available for the surgical treatment of CRC, which will show their utility in improving treatment outcome in the future. Although promising in their pilot effort, the AI applications mentioned in this article are not ready yet for large-scale clinical usage.

Autonomous robots are still part of the future, but the moment they will become part of the surgical treatment is getting nearer. The hardware part is available (commercially available surgical robots), while several intraoperative aspects of CRC surgeries have been captured, analyzed and successfully reproduced using AI.

The current use of AI to the medical area is steadily changing the diagnostic and treatment approach to a wide range of diseases. While many AI applications have been used and investigated in several cancer entities, such as lung and breast cancer, the use of AI in CRC is still in its early stages (44). AI’s utility in CRC has been established mostly for aiding in screening and staging. Meanwhile, evidence on the use of AI in colorectal surgery is limited.

Surgical data and applications are more difficult to analyze and use than data for AI in screening endoscopy, radiology, and pathology. Surgical movies are dynamic, displaying difficult-to-model tool-tissue interactions that modify and even entirely reshape anatomical situations. Surgical workflows and techniques are difficult to standardize, particularly in long and unpredictable operations like colorectal surgery for CRC. During surgical interventions, surgeons use prior knowledge, such as preoperative imaging, as well as their personal experience and intuition to make decisions. More and better data are required to address these challenges. This includes reaching an agreement on annotation techniques (45) and publicly publishing vast, high-quality annotated datasets. Multiple institutions must collaborate in this context to ensure that data are diverse and representative (46). Such datasets will be critical for training stronger AI models, and also for demonstrating generalizability through external validation studies (47).

## 5 Conclusions

The use of AI in CRC surgery is still at its beginnings, despite the fact that AI has already demonstrated its clear clinical benefits in the screening and diagnosis of CRC. Many studies are still in the preclinical phase. The development of AI models capable of reproducing a colorectal expert surgeon’s skill, the creation of large and complex datasets and the standardization of surgical colorectal procedures will contribute to the widespread use of AI in CRC surgical treatment.

## Author contributions

MA contributed to the conception, research of the primary literature, and writing of the article. DL. MM contributed to the conception and research of the primary literature for the article. SO contributed to the conception, research of the primary literature, and writing of the article. All authors contributed to the article and approved the submitted version.
